# Environmental Forcing of Nitrogen Fixation in the Eastern Tropical and Sub-Tropical North Atlantic Ocean

**DOI:** 10.1371/journal.pone.0028989

**Published:** 2011-12-13

**Authors:** Micha J. A. Rijkenberg, Rebecca J. Langlois, Matthew M. Mills, Matthew D. Patey, Polly G. Hill, Maria C. Nielsdóttir, Tanya J. Compton, Julie LaRoche, Eric P. Achterberg

**Affiliations:** 1 School of Ocean and Earth Science, National Oceanography Centre Southampton, University of Southampton, Southampton, United Kingdom; 2 Leibniz Institute for Marine Sciences, Kiel, Germany; 3 Stanford University, Department of Geophysics, Stanford, California United States of America; 4 National Institute of Water and Atmospheric Research, Hamilton, New Zealand; Netherlands Institute of Ecology, The Netherlands

## Abstract

During the winter of 2006 we measured *nifH* gene abundances, dinitrogen (N_2_) fixation rates and carbon fixation rates in the eastern tropical and sub-tropical North Atlantic Ocean. The dominant diazotrophic phylotypes were filamentous cyanobacteria, which may include *Trichodesmium* and *Katagnymene*, with up to 10^6^ L^−1^ *nifH* gene copies, unicellular group A cyanobacteria with up to 10^5^ L^−1^ *nifH* gene copies and gamma A proteobacteria with up to 10^4^ L^−1^ *nifH* gene copies. N_2_ fixation rates were low and ranged between 0.032–1.28 nmol N L^−1^ d^−1^ with a mean of 0.30±0.29 nmol N L^−1^ d^−1^ (1σ, n = 65). CO_2_-fixation rates, representing primary production, appeared to be nitrogen limited as suggested by low dissolved inorganic nitrogen to phosphate ratios (DIN:DIP) of about 2±3.2 in surface waters. Nevertheless, N_2_ fixation rates contributed only 0.55±0.87% (range 0.03–5.24%) of the N required for primary production. Boosted regression trees analysis (BRT) showed that the distribution of the gamma A proteobacteria and filamentous cyanobacteria *nifH* genes was mainly predicted by the distribution of *Prochlorococcus*, *Synechococcus*, picoeukaryotes and heterotrophic bacteria. In addition, BRT indicated that multiple a-biotic environmental variables including nutrients DIN, dissolved organic nitrogen (DON) and DIP, trace metals like dissolved aluminum (DAl), as a proxy of dust inputs, dissolved iron (DFe) and Fe-binding ligands as well as oxygen and temperature influenced N_2_ fixation rates and the distribution of the dominant diazotrophic phylotypes. Our results suggest that lower predicted oxygen concentrations and higher temperatures due to climate warming may increase N_2_ fixation rates. However, the balance between a decreased supply of DIP and DFe from deep waters as a result of more pronounced stratification and an enhanced supply of these nutrients with a predicted increase in deposition of Saharan dust may ultimately determine the consequences of climate warming for N_2_ fixation in the North Atlantic.

## Introduction

Nitrogen is a key nutrient, limiting primary production throughout much of the world's upper oceans [1]. In tropical and sub-tropical oligotrophic oceanic environments, biological fixation of dinitrogen (N_2_) provides an important source of new nitrogen for primary production and carbon export [2]. Nevertheless, our knowledge of the diversity, abundance and distribution of diazotrophs (N_2_ fixing micro-organisms) is limited and the factors that control N_2_ fixation in the marine environment are still poorly understood [3].

Until recently, the majority of N_2_ fixation studies in the Atlantic Ocean have focused solely on *Trichodesmium* [4]. However, with the increasing application of molecular genetic analyses, more information is becoming available on the diversity, abundance and distribution of diazotrophs [5], [6]. It is now clear that a broad suite of diazotrophs inhabit the oceans including diatom endosymbionts, *Crocosphaera* and other uncultured small unicellular diazotrophs, e.g. group A and C cyanobacteria, gamma A proteobacteria, and cluster III *nifH* phylotypes [5], [7], [8], [9]. Furthermore, large scale patterns in the distribution of diazotrophs are recognized [10]. In the western part of the North Atlantic Ocean *Trichodesmium* biomass and N_2_ fixation rates are reported to be high [4], [10], whereas in the eastern part of the North Atlantic N_2_-fixing unicellular cyanobacteria are reported to be responsible for a significant part of the N_2_ fixation [3], [11], [12].

Several environmental factors have been reported to control N_2_ fixation by *Trichodesmium*. Laboratory experiments and field observations suggest that N_2_ fixation by *Trichodesmium* is limited to water temperatures between 20°C–32°C [13]. However, co-variation of low oxygen, low nutrients and high light, due to a more strongly stratified water column, could underlie the influence of temperature on N_2_ fixation [14]. N_2_ fixation in natural populations dominated by *Trichodesmium* can be controlled by phosphorus or iron availability [15], [16], [17] or both [18]. Additionally, enhanced concentrations of the nutrients nitrate (NO_3_^-^), ammonium and organic nitrogen sources including urea and glutamate may inhibit N_2_ fixation of *Trichodesmium* [19], [20], [21].

By contrast the environmental factors that control the abundance and activity of unicellular diazotrophs are unknown. Langlois et al. (2008) reported that most diazotrophic phylotypes in the North Atlantic Ocean are almost completely restricted to regions with NO_3_^-^ concentrations <0.5 µM and are limited to warmer seawater temperatures (20–30°C). The majority of the diazotrophic community in general and the uncultured group A unicellular cyanobacteria in particular, have been observed in regions with seawater temperatures of about 22°C. The uncultured γ-proteobacterium A group (gamma A) and filamentous group (*Trichodesmium* spp.) have been observed in conditions with mean temperatures of ca. 24 and 25°C, respectively [3]. However, N_2_ fixation by marine unicellular diazotrophs at seawater temperatures as low as 15–19°C has been reported [22], [23].

The tropical and sub-tropical North Atlantic Ocean, in the vicinity of the Cape Verde islands, is a region receiving enhanced Saharan dust inputs [24] and some of the highest N_2_ fixation rates and diazotroph abundances have been reported here [3], [4], [10], [11], [25], [26]. To investigate which environmental variables determine the distribution of the dominant diazotrophs and N_2_ fixation rates in this area we measured *nifH* gene abundance, N_2_ fixation and CO_2_ fixation rates, and an extensive set of environmental variables including nanomolar concentrations of phosphate (PO_4_^3-^) and NO_3_^-^ + NO_2_^-^ (hereafter termed NO_3_^-^), dissolved Fe (DFe) and dissolved Al (DAl) as a proxy of dust inputs, and a diverse set of biological variables. We used boosted regression trees analysis (BRT) to investigate the relative contribution of the environmental variables in explaining the distribution of diazotrophs and the rates of N_2_ fixation.

## Materials and Methods

### Sampling

**Figure 1:**
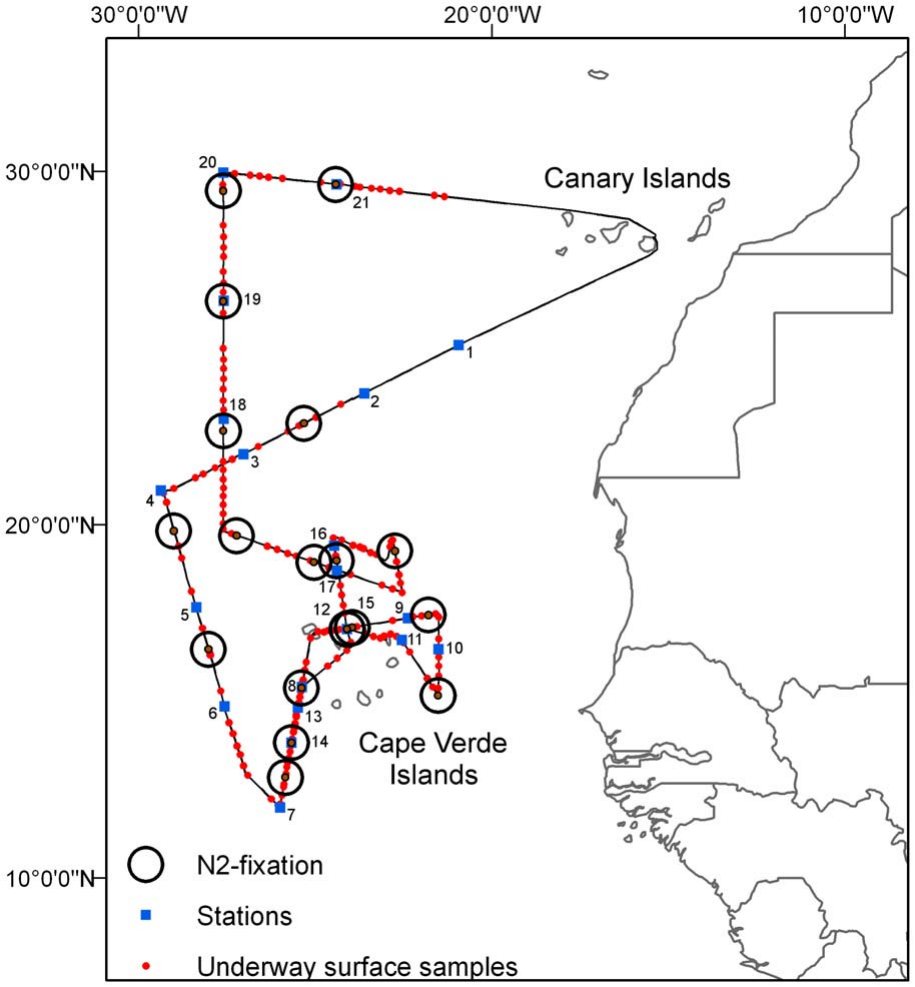
The cruise track of the P332 cruise in January-February 2006.

This study was conducted during a cruise in the vicinity of the Cape Verde islands (26 January to 26 February 2006) on board the research vessel *FS Poseidon* (cruise P332) (Fig. 1). Surface seawater was pumped into a trace metal clean laboratory container using a Teflon diaphragm pump (Almatec A-15, Germany) connected by an acid-washed braided PVC tubing to a towed fish positioned at ca. 3 m depth alongside the ship. Unfiltered seawater was collected for N_2_ fixation measurements in an acid-cleaned 25 L low density polyethylene carboy (Nalgene). Simultaneously, material was collected by vacuum filtration (0.2 bar) of 1.5–2 L onto 0.22 µm Durapore (Millipore) filters for analysis of *nifH* genes. Within 10 minutes after sampling, these filters were stored at −80°C until extraction in the laboratory. Samples for analysis of DFe and DAl, Fe-binding ligands, and nanomolar PO_4_^3-^ and NO_3_^-^ were collected from the towed fish and filtered in-line using a filter capsule (Sartorius, Sartobran 300) with a 0.2 µm filtration cut-off. Samples for total dissolved phosphate (TDP), total dissolved nitrogen (TDN) and ammonium (NH_4_^+^) were sampled from the surface water bottles of the CTD rosette frame. Oxygen concentrations were measured using a Seabird sensor on the CTD frame. Photosynthetic variables were measured using active fluorescence measurements performed by fast repetition rate fluorometry (FRRF) using a Chelsea Instruments FASTtracka FRRF (Chelsea Technologies Group, UK) mounted on the CTD frame. Samples for Chlorophyll *a* (Chl *a,* 0.7 µm GF/F filtered and stored at −80°C), dissolved silicate (SiO_4_^4-^) and flow cytometry were taken from the non-toxic underway surface seawater supply (sampling depth ca. 4 m). Underway temperature and salinity were determined using a thermosalinograph (Meerestechnik Elektronic, Germany).

### N_2_ and C fixation rates

Rates of N_2_ fixation (^15^N uptake) and CO_2_ fixation (^13^C uptake) were measured using the stable isotopes of N (^15^N_2_) and C (H^13^CO_3_^-^) [27]. All collection and incubation steps were carried out using trace-metal clean techniques. N_2_- and CO_2_ fixation rates were determined in an unfiltered, non-concentrated homogeneous surface seawater sample collected in a 25 L carboy, and subsequently incubated in quadruplicate in gas tight 4 L polycarbonate bottles (Nalgene) equipped with Teflon lined butyl septum caps. Any air bubbles were removed from the bottles following the filling process. Trace quantities of ^15^N_2_ (99 atom %, Cambridge Isotope Laboratories) were added (4 mL, 9% of ambient N_2_) using a gas-tight syringe (Chromatographie-Zubhör, Germany). The H^13^CO_3_^-^ solution (99 atom %, Brand company) was added to a final concentration of 25 µM or 1.2% of ambient TCO_2_ as H^13^CO_3_^-^ [16]. Incubations were performed on deck and the temperature was controled with circulation of surface seawater. At night, incubators were covered with a black plastic sheet to protect the incubations against the influence of the ship's lights and during daytime the light was attenuated to 20% surface irradiance using blue filters (Lagoon blue #172, Lee Filters, UK). Underway seawater samples were typically collected in the morning and evening but on occasions in the afternoon. Samples were typically incubated for a minimum of 24 h and a maximum of 31.8 h.

All incubations were terminated by on-board filtration under gentle vacuum through a pre-combusted GF/F filter (Whatman, UK). Filters were frozen (−20°C) and stored for further analysis. At the home laboratory the filters were dried at 60°C, acid-fumed with concentrated HCl to remove carbonates, and then stored over desiccant until analysis. Each filter was packed into a tin capsule and pelletized for elemental and isotopic analysis.

Carbon and nitrogen total mass and isotope ratios were measured at the Stable Isotope Laboratory at Stanford University, Stanford, CA (USA) using a continuous-flow mass spectrometer (Finnegan Delta Plus XL, Germany), coupled to an elemental analyzer (Costech ECS 4010, USA). Acetanilide was used as a mass calibration standard. Isotope values were calibrated using international carbon (IAEA-CH-6) and nitrogen (IAEA-NO-3) reference materials with assigned δ^ 13^C and δ^ 15^N values of −10.4‰ and +4.7‰. Blank corrections were made following [28]. Analytical precision (1 standard deviation) was±0.2‰ for δ^15^N and±0.1‰ for δ^13^C. Carbon and nitrogen isotope ratios are reported in ‰ relative to Vienna–PeeDee belemnite for δ^13^C and atmospheric N_2_ for δ^15^N. The N_2_- and CO_2_ fixation rates were calculated by isotope mass balance [27]. The solubility of N_2_ in seawater was calculated according to Hamme and Emerson [29]. Removal of outliers (1.5 times below or above the first and third quartile, respectively) resulted in 65 N_2_ fixation rates and combined with missing values in 54 carbon fixation rates at 18 stations.

Reported averages are accompanied by “n”, where “n” is either the number of individual incubation bottles (bottles) in which the variable was measured for each replicate bottle or the number of experiments (experiments) where the same value for an environmental variable was given to all replicates of one experiment.

### Nutrients, trace metals and environmental data

Values for DFe and the organic complexation of Fe were taken from Rijkenberg et al. [30]. Dissolved Al (<0.2 µm) was determined using the fluorometric lumogallion method with a spectrofluorometer (model Aminco, American Instruments Co.) [31]. NO_3_^-^ and PO_4_^3-^ were measured at nanomolar concentrations using a system comprised of a conventional segmented-flow autoanalyser connected to two 2-metre liquid waveguide capillary cells (WPI Inc, USA) and using miniaturized spectrophotometric systems (Ocean Optics Inc., USA) [32]. NO_3_^-^ and PO_4_^3-^ were determined colorimetrically using the sulphanilamide-NEDD and molybdenum blue methods, respectively, achieving detection limits of 1.5 nmol L^−1^ NO_3_^-^ and 0.8 nmol L^−1^ PO_4_^3-^ [32]. Micromolar concentrations of silicate were measured on a Scalar Sanplus autoanalyser [33]. Ammonium was measured at nanomolar concentrations based on the reaction of NH_4_^+^ with orthophtaldialdehyde in the presence of sulphite [34]. Reagents were added immediately after collection of the samples and fluorometric analysis (excitation at 370 nm and emission at 420 nm) was conducted on a Turner Design fluorometer (TD700) after a 24 hours incubation.

Seawater samples for measurement of TDN were filtered using combusted (450 °C, 4–6 h) glass-fibre filters (Whatman, GF/F). The filtrate was transferred to a combusted (450 °C, 4–6 h) glass ampoule and stabilised by acidification to pH 2 using hydrochloric acid and subsequently flame-sealed. The ampoules were stored at 4°C until analysis. TDN was measured using high-temperature combustion on a Shimadzu TOC 5000A total carbon analyser (Shimadzu Corp, Japan) coupled with a Sievers NCD 255 nitrogen chemiluminescence detector (Sievers Instruments, Inc, US) [35]. DON concentrations were calculated by subtracting the ammonium and NO_3_^-^ + NO_2_^-^ concentrations from the TDN concentrations.

Seawater samples for total dissolved phosphorus (TDP) were filtered using glass-fibre filters (Fisherband® MF 300; nominal pore size 0.7 µm) and subsequently irradiated using a UV lamp to oxidise organic phosphorus compounds to PO_4_^3-^ which was subsequently measured by colorimetry using a Skalar Sanplus autoanalyser according to Kirkwood [33]. Dissolved organic phosphorus (DOP) was calculated by subtracting phosphate concentration from the measured TDP.

Chl *a* was determined on duplicate 500 mL seawater samples filtered through 25 mm diameter glass-fiber filters (Fisherbrand MF 300). Filters were frozen at −80°C until onboard analysis by fluorometry. Samples were extracted in 7 mL of 90% acetone for 24 h at 4°C; Chl *a* concentrations (>0.7 µm) in the extracts were measured using a TD-700 Turner Designs fluorometer following calibration with fresh Chl *a* standard (Sigma, UK).

Seawater samples of 1.6 mL were fixed with a final concentration of 1% paraformaldehyde for 24 hours at 4°C and subsequently frozen at −80°C until processed by flow cytometry (FACSCalibur, Becton Dickinson, BD Biosciences, Oxford, UK). Two groups of cyanobacteria, *Prochlorococcus* spp. and *Synechococcus* spp, and a broad group of picoeukaryotes were identified by their characteristic autofluorescence [36]. The heterotrophic prokaryotes (Bacteria and Archaea) were counted after staining of the whole microbial population with the nucleic acid stain SYBR Green I and subsequent subtraction of the autofluorescent cells [36].

### qPCR

The *nifH* abundances of filamentous (*Trichodesmium* spp.), unicellular Group A, and Gamma A diazotrophs were determined using TaqMan primers and probe sets as described in Langloiset al. (2008). qPCR reactions contained 1x TaqMan master mix (Applied Biosystems), 40 ng µl^−1^ bovine albumin (BSA), 5 pmol µL^−1^ each forward and reverse primers, 100 nmol L^−1^ TaqMan probe, and 5 µl DNA (average of 4 ng DNA). Plasmid standards, described in Langlois et al. (2008), were run in duplicate, as were template controls (NTC). Samples were run in triplicate. All reactions were run on an ABI Prism 7000 instrument (Applied Biosystems) using the default cycling program with 45 cycles. Raw data were analyzed using the ABI 7000 system SDS software (version 1.2.3) with RQ study application. Primer amplification efficiencies were 97% for filamentous and Group A and 95% for Gamma A; calculated using the formula E = 10^−1/slope^-1 [37]. No amplification was observed in the NTCs, thus setting the potential detection limit to 1 copy L^−1^. When the elution and filtration volumes were accounted for the actual detection limit was 50 copies L^−1^.

### Environmental variables

Environmental variables were either collected at the time of sampling or taken from the nearest stations or underway sample points. The environmental variables available for analysis included: i) oceanographic variables such as oxygen, salinity, the water-mass as identified by a temperature salinity plot, and the mixed layer depth [25], ii) as factor the dust event on 3&4 Feb 2006, iii) NO_3_^-^, PO_4_^3-^, SiO_4_^4-^, NH_4_^+^, DON and DOP, iv) biological variables including the photosynthetic efficiency, F_v_/F_m_, and the PSII cross-section (σ_PSII_), flow cytometer counts of heterotrophic prokaryotes, picoeukaryotes, *Prochlorococcus*, *Synechococcus* and the *nifH* gene abundance of gamma A proteobacteria, unicellular group A and filamentous cyanobacteria, and the N_2_ fixation rates, and v) trace metals DAl and DFe, free and total concentrations of Fe-binding ligands and their conditional stability constant log K'. Incubation time and the time of ^15^N_2_-tracer addition were also considered in the model to check whether these influenced the N_2_ fixation rate response variable.

### Boosted regression trees analysis (BRT)

BRT was used to identify which environmental variables could describe (1) log *nifH* gene abundances of gamma A proteobacteria, (2) filamentous and group A cyanobacteria, (3) total *nifH* gene abundance (sum of group A proteobacteria, gamma A and filamentous cyanobacteria), and (4) N_2_ fixation rates [38], [39]. As the data was continuous and normally distributed, the model was fit using a Gaussian error distribution and link function [38]. BRT is a relatively new statistical technique and is based on a combination of regression trees and boosting. Boosting increases the emphasis on poorly modelled observations and iteratively fits regression trees to the data. BRT has advantages over standard techniques in that it can fit complex non-linear relationships (Elith et al. 2008) and can deal with missing values. Following the model simplification procedure of Elith et al. (2008), we identified the variables that provided the best model performance. Model performance was assessed using a Pearson's correlation and deviance measures, i.e. goodness of fit (Elith et al. 2008). All evaluation statistics were calculated with 6-fold cross- validation and the BRT models were run 30 times to ensure stable estimates of model evaluation (Elith et al. 2008). Replicates of experimental treatments were placed into a single fold so that the models could be evaluated on independent treatment data. All BRT models were fitted in R (v2.6.0, www.Rproject.org) using the ‘gbm’ library [40].

## Results and Discussion

### Study area

**Figure 2:**
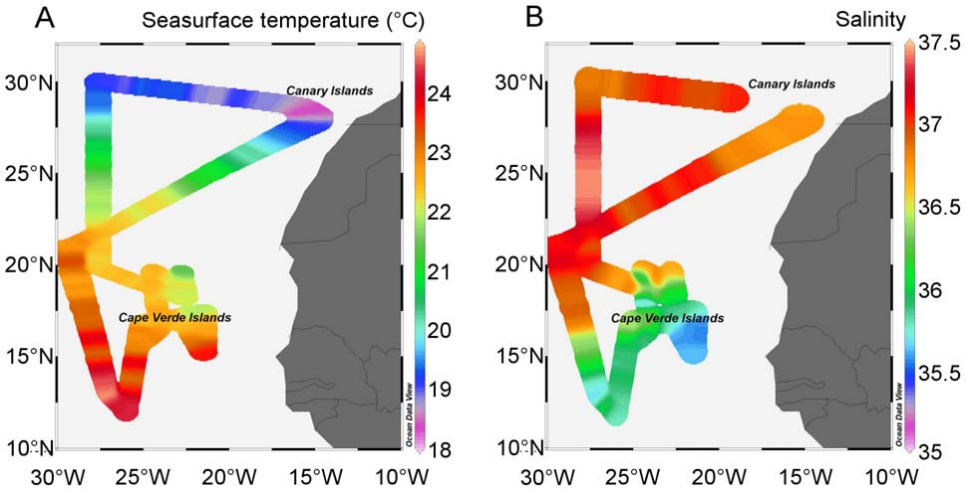
The sea surface (A) temperature (°C) and (B) salinity during the P332 cruise.

Our study area was situated between 12–30°N and 20–30°W, with most of the N_2_- and CO_2_ fixation experiments performed in proximity of the Cape Verde islands. The surface waters to the north and south of the Cape Verde islands consisted of the westward flowing North Equatorial Current (NEC) and North Equatorial Counter Current (NECC), respectively [41]. The surface seawater temperature varied between 18°C in the vicinity of the Canary Islands and up to 24.8°C at ∼12°N, south of the Cape Verde islands (Fig. 2A). The salinity of the surface seawater varied between 35.62 and 37.36 southeast and northwest of the Cape Verde islands, respectively (Fig. 2B).

### Nitrogen fixation rates and primary productivity

**Figure 3:**
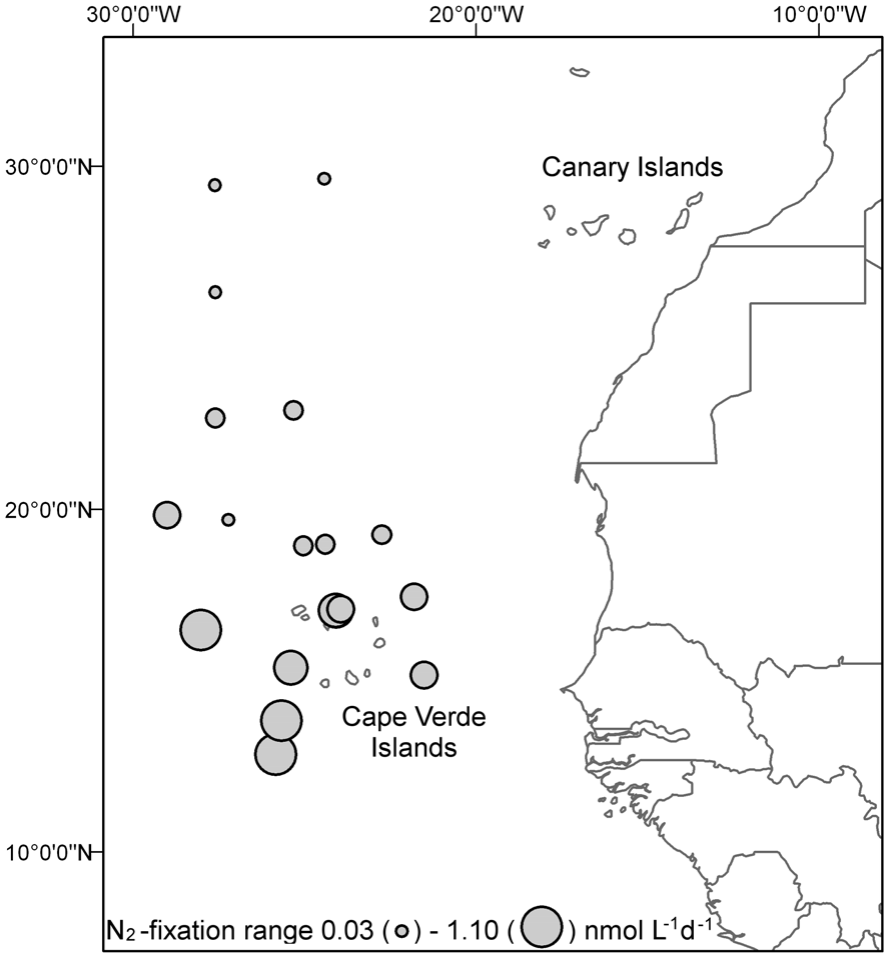
Bubble plot of the average N_2_ fixation rates (nmol L^−1^ d^−1^) based on 3 to 4 replicates.

**Figure 4:**
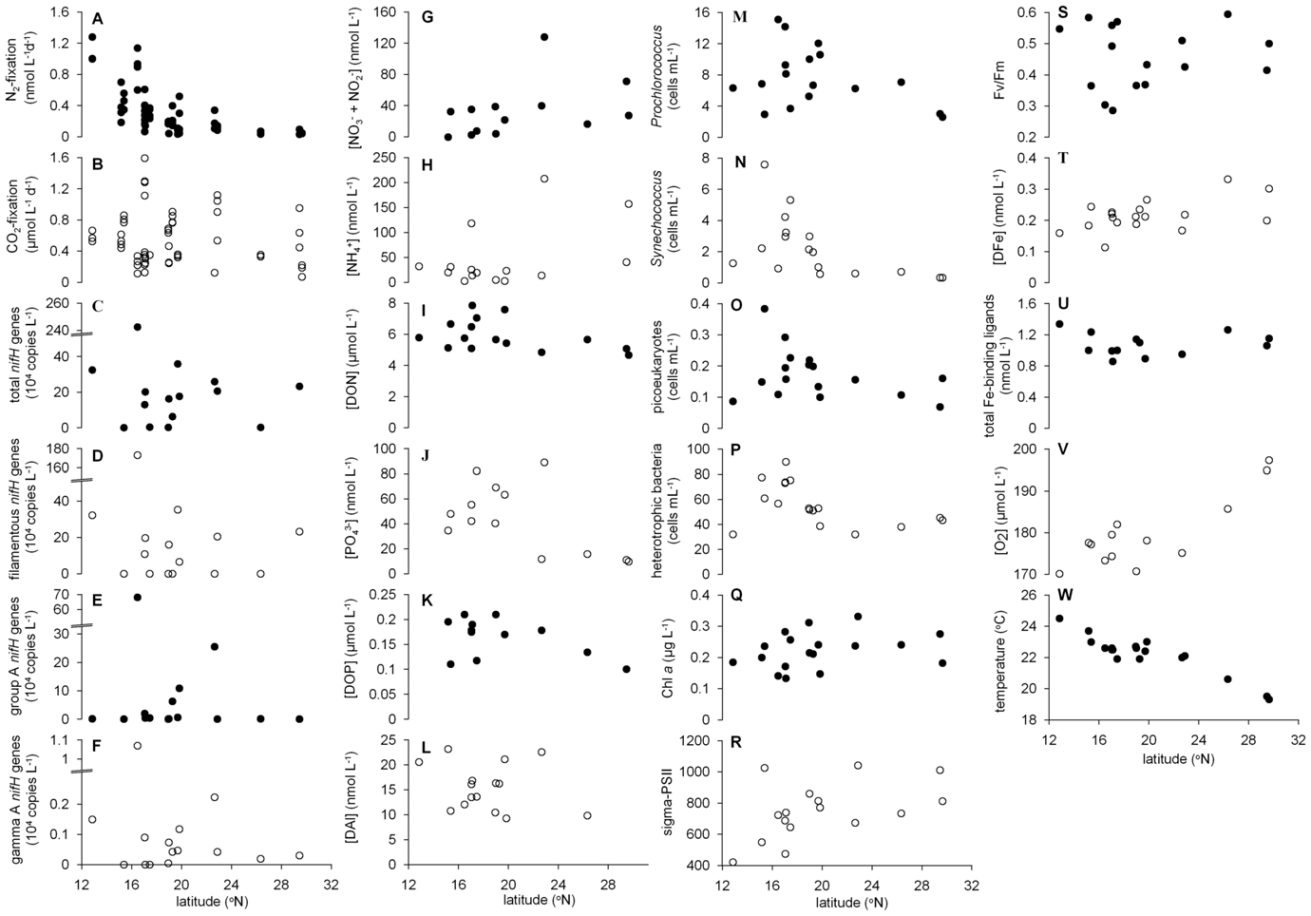
The environmental parameters measured during the P332 cruise in the Cape Verde region. The (A) N_2_ fixation rate, (B) CO_2_ fixation rate, (C) total *nifH* gene abundance, (D) filamentous *nifH* gene abundance, (E) group A *nifH* gene abundance, (F) gamma A *nifH* gene abundance, (G) NO_3_^-^ + NO_2_^-^, (H) NH_4_^+^, (I) DON, (J) PO_4_^3-^, (K) DOP, (L) DAl, (M) *Prochlorococcus* spp. abundance, (N) *Synechococcus* spp. abundance, (O) picoeukaryote abundance, (P) heterotrophic prokaryote abundance, (Q) Chl *a*, (R) σ_PSII_, (S) F_v_/F_m_, (T) DFe, (U) total Fe-binding ligands, (V) O_2_, (W) temperature as a function of latitude.

Winter N_2_ fixation rates ranged between 0.032–1.28 nmol N L^−1^ d^−1^ with an overall mean of 0.30±0.29 nmol N L^−1^ d^−1^ (n = 65 bottles) (Fig. 3). N_2_ fixation rates were lowest north and east of the Cape Verde islands (0.21±0.15 nmol N L^−1^ d^−1^, range 0.032–0.70 nmol N L^−1^ d^−1^, n = 55 bottles) and highest to the south and west of the Cape Verde islands (0.91±0.31 nmol N L^−1^ d^−1^, range 0.35–1.28 nmol N L^−1^ d^−1^, n = 10 bottles) (Fig. 3 and 4A). Using acetylene reduction assays, Staal et al. [12] also observed low winter time N_2_ fixation rates of 0.154±0.091 nmol N L^−1^ d^−1^ in light controlled (200 µm m^−2^ s^−1^) incubations southeast of the Cape Verde islands. Autumn N_2_ fixation rates between 2.4–151.2 nmol N L^−1^ d^−1^ (non-size fractionated and non-trace metal clean sampled) [26] have been reported for the same study region. Unicellular N_2_ fixation rates of 3.48 nmol N L^−1^ d^−1^ have been reported in other regions of the tropical and sub-tropical North Atlantic Ocean [42]. Whereas Voss et al. [11] reported occasional high rates of N_2_ fixation of up to 75 nmol N L^−1^ d^−1^ in surface waters (non-size fractionated and non-trace metal clean sampled during a high dust deposition event in autumn) along 10°N in the eastern North Atlantic, though most values were below 6 nmol N L^−1^ d^−1^. The higher N_2_ fixation rates as measured by Voss et al. [11] coincided with enhanced N_2_ fixation rates and diazotroph abundance along 10°N as reported by Moore et al. [16] and Staal et al. [12]. N_2_ fixation rates in the eastern Atlantic Ocean along ca. 10°N may be higher as a result of warmer seawater temperatures, lower oxygen concentrations and a higher supply of Fe and phosphate due to Saharan dust inputs [16]. However, differences in season and location may complicate direct comparison of N_2_ fixation rates.

Primary productivity was measured in the same bottles as N_2_ fixation and ranged between 0.07–1.59 µmol C L^−1^ d^−1^ with a mean of 0.55±0.34 µmol C L^−1^ d^−1^ (n = 54 bottles) (Fig. 4B). Little latitudinal variation in rates of primary production was observed as seen for N_2_ fixation rates. Converting N_2_ fixation to C-uptake rates via Redfield equivalents showed that the contribution of N_2_ fixation to primary production was between 0.03–5.24%, with an average of 0.55±0.87% (n = 54 bottles). In our study, the average contribution of N_2_ fixation to primary production was substantially lower than the values of 5.8–12.2% reported by Voss et al. [11]. Metabolic control of N_2_ fixation by other nitrogen sources during our study was unlikely. Surface water NO_3_^-^ concentrations ranged between 1.5–128 nmol L^−1^ (Fig. 4G; n = 13 experiments) and were well below the concentrations (>0.5 µmol L^−1^) reported to inhibit N_2_ fixation [20], [43]. The ammonium concentrations ranged between 2.3–208 nmol L^−1^ (n = 15 experiments) and DON between 4.7–7.8 µmol L^−1^ (n = 15 experiments) (Fig. 4H–I). Fixed nitrogen sources such as urea or ammonium can inhibit N_2_ fixation in *Trichodesmium* [19], although in the laboratory *Trichodesmium* has been reported to regulate ammonium metabolism and N_2_ fixation within its circadian rhythm [44]. Calculated from primary production and compensated for the nitrogen provided by N_2_ fixation, the turnover time of the DIN pool (DIN = NO_3_^-^ + NO_2_^-^ + NH_4_^+^) was less than 1 day in 19 out of 33 incubations. This implies that the DIN pool including N_2_-fixed nitrogen did not provide sufficient nitrogen to sustain the observed primary production. Access to DON or rapid recycling of nitrogen were likely required for provision of additional nitrogen sources [45].

The PO_4_^3-^ concentrations of the surface water used in our N_2_ fixation incubations ranged between 10–92 nmol L^−1^, with a mean of 44±27 nmol L^−1^ (Fig. 4J; n = 13 experiments). Surface dissolved inorganic phosphate concentrations below 1 nmol L^−1^ were reported in the western North Atlantic Ocean, with DIN:DIP ratios between 20–30, suggesting that PO_4_^3-^ may control primary production and N_2_ fixation rates [46]. However, very low inorganic DIN:DIP ratios with a mean of 2±3.2 (n = 94, range 0–15) in the vicinity of the Cape Verde islands suggest that nitrogen, depending on the availability of DON and nitrogen recycling rates, was the primary limiting nutrient [16], [18]. Indeed, in our N_2_ fixation incubations the turnover time of the dissolved inorganic phosphate pool (DIP) based on primary production varied between 1.2–36 days (n = 40 bottles). Primary production and N_2_ fixation in our incubations were unlikely to be controlled by the availability of PO_4_^3-^. Based on the conversion of primary production to PO_4_^3-^ demands via a C:P Redfield ratio of 106, PO_4_^3-^ concentrations were neither limiting primary production anywhere else in the vicinity of the Cape Verde islands (mean 39±24 nmol L^−1^ PO_4_^3-^ (n = 96), range 10–100 nmol L^−1^).

The DFe concentrations in the surface waters were on average 0.22±0.05 nmol L^−1^ (n = 18 experiments) and ranged between 0.11 and 0.33 nmol L^−1^. The Fe requirement for primary production, using a conversion of 60.5 µmol Fe per mol of fixed C as based on average phytoplankton nutrient requirements [47], varied between 4.0 10^−3^–0.1 nmol L^−1^ DFe (n = 54 bottles). Thus the DFe concentrations were 2.3–73 times higher than the calculated Fe requirements. Conversion of N_2_ fixation rates using Redfield stoichiometry into C-uptake according to Voss et al. [11], assuming 28 µmol Fe per mol of fixed C for phototrophic diazotrophs as based on *Trichodesmium* [48], yield Fe requirements between 6.0 10^−6^–2.4 10^−4^ nmol L^−1^ Fe (n = 65 bottles) which are ca. 10^4^ times lower than the DFe concentrations. Iron:C ratios as high as 450±242 µmol Fe per mol C have been reported for *Trichodesmium* in Australian waters [15] resulting in Fe requirements between 1.0 10^−4^–3.8 10^−3^ nmol Fe (n = 65 bottles) in our incubations, suggesting that even using high Fe:C ratios there still is approximately a 600 fold surplus of DFe. These calculations indicate that diazotrophic activity itself will not induce Fe limitation. However, the large uncertainties in Fe:C ratios which vary with region, growth conditions, phytoplankton population [49], and uncertainties in the biological availability of Fe [50] make it difficult to assess (micro)nutrient control of diazotrophy. In fact, along 10°N in the eastern tropical North Atlantic, Voss et al. [11] determined that Fe concentrations were higher than the estimated requirement for diazotrophy, while at the same time Mills et al. (2004) showed that Fe (in combination with P) stimulated N_2_ fixation rates in this region. Over 99% of the DFe fraction in the vicinity of the Cape Verde islands was complexed to organic Fe-binding ligands [30]. It is still unknown what fraction of this organically complexed Fe pool is directly or indirectly available for uptake by phytoplankton and diazotrophs [50], [51]. Clearly, calculated quotas and stoichiometric ratios may not apply as well as we assume and more detail regarding the availability to diazotrophs of the organically complexed Fe is required.

### Environmental variables predicting nifH gene abundance and N_2_ fixation rates

**Figure 5:**
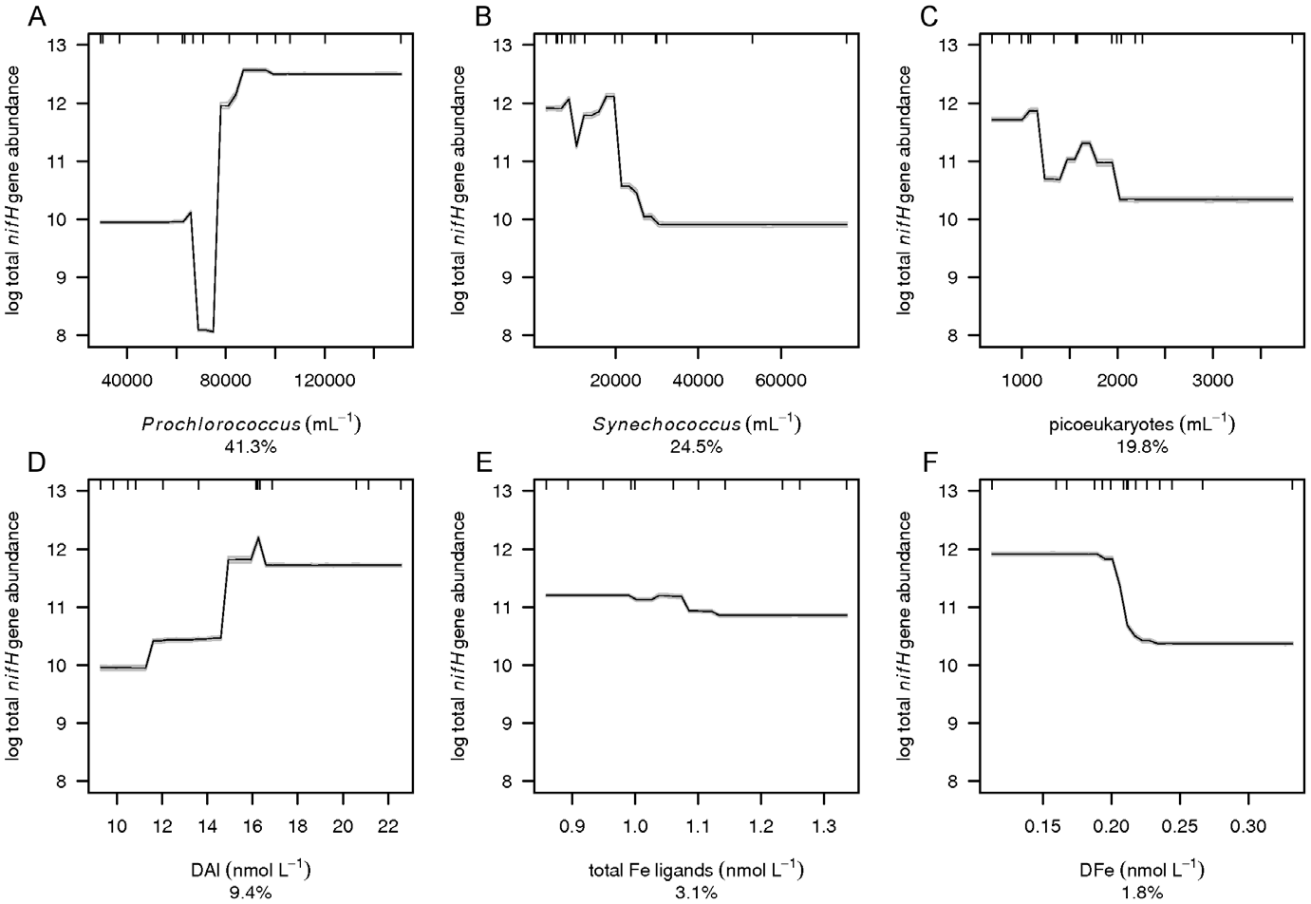
Mean partial dependence plots for the environmental variables describing the total *nifH* gene abundance. The partial dependence plots with 95% confidence intervals (light grey, based on bootstrapping of 30 BRT runs and indicating robustness of model performance) for the 6 environmental variables best at explaining the variation in total *nifH* gene abundance (the sum of Group A, gamma A and filamentous *nifH* genes). The 6 environmental variables are A) *Prochlorococcus*, B) *Synechococcus*, C) picoeukaryotes, D) DAl, E) total Fe ligands and F) DFe. The y-axis is centered to have zero mean over the log distribution of the fitted total *nifH* gene abundance. A common scale is used for the x-axis. Rug plots at inside top of plots show the distribution of sites across that variable, in deciles.

BRT analysis was used to investigate which environmental variables explain the distribution of group A, gamma A, filamentous and total *nifH* gene abundances (the sum of group A, gamma A and filamentous *nifH* gene abundances) as well as N_2_ fixation rates. The group A *nifH* gene abundance was poorly described by the BRT analysis and is therefore not further discussed. However, BRT performed well for the gamma A and filamentous phyloptypes as well as the total *nifH* gene abundance and N_2_ fixation rates with respective cross validated Pearson correlations of 0.94, 0.53, 0.98 and 0.47 (Table 1) and robust model performance for a relatively small data set as shown by small 95% confidence intervals in the mean partial dependence plots based on bootstrapping of 30 BRT runs (Fig. 5–8).

**Figure 6 pone-0028989-g006:**
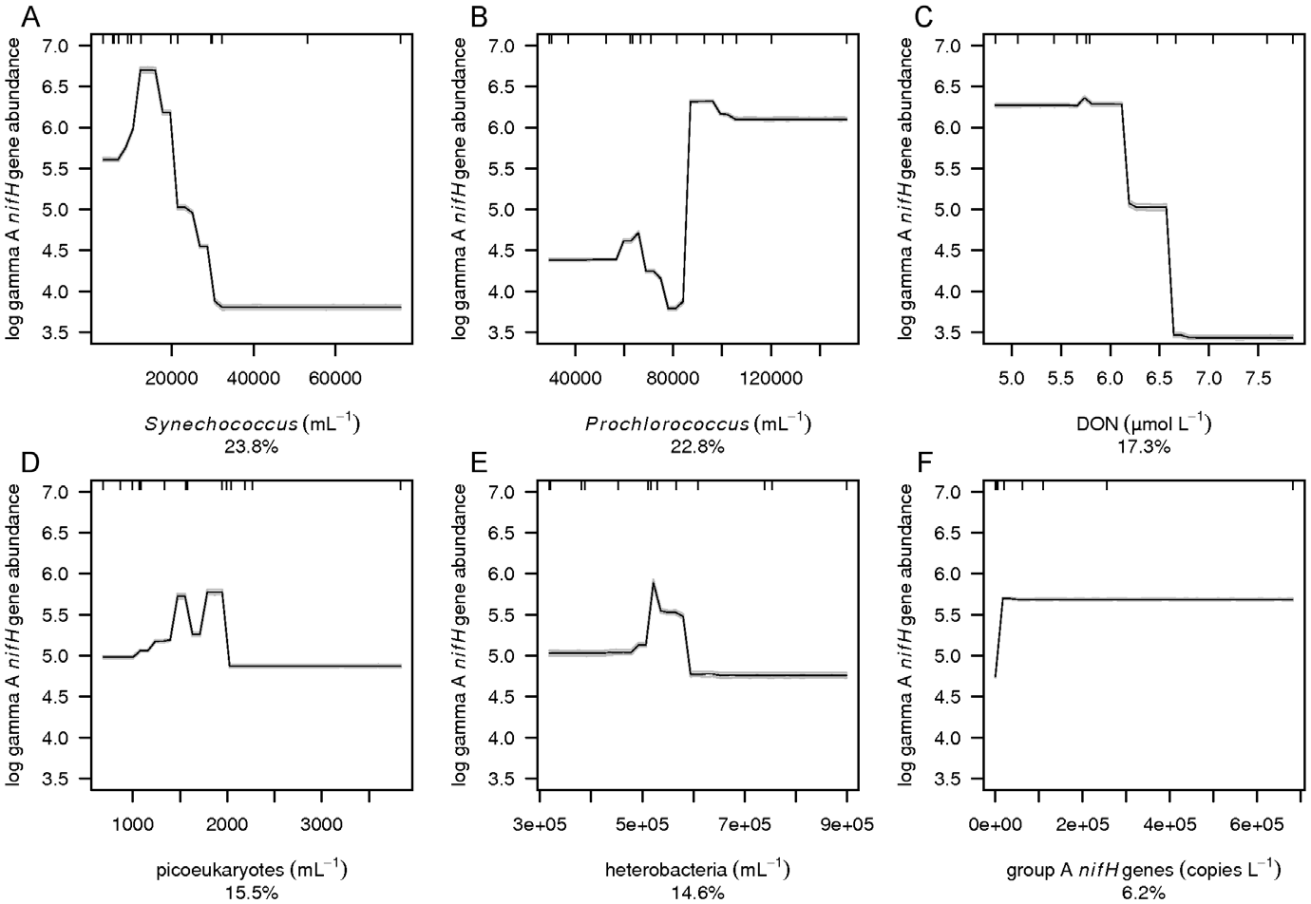
Mean partial dependence plots for the environmental variables describing the gamma A *nifH* gene abundance. Mean partial dependence plots with 95% confidence intervals (light grey, based on bootstrapping of 30 BRT runs and indicating robustness of model performance) for the 6 environmental variables best at explaining the variation in gamma A *nifH* gene abundance. The 6 environmental variables are A) *Prochlorococcus*, B) *Synechococcus*, C) heterotrophic prokaryotes, D) picoeukaryotes, E) DON and F) group A *nifH* genes. The y-axis is centered to have zero mean over the log distribution of the fitted gamma A *nifH* gene abundance. A common scale is used for the x-axis. Rug plots at inside top of plots show the distribution of sites across that variable, in deciles.

**Figure 7 pone-0028989-g007:**
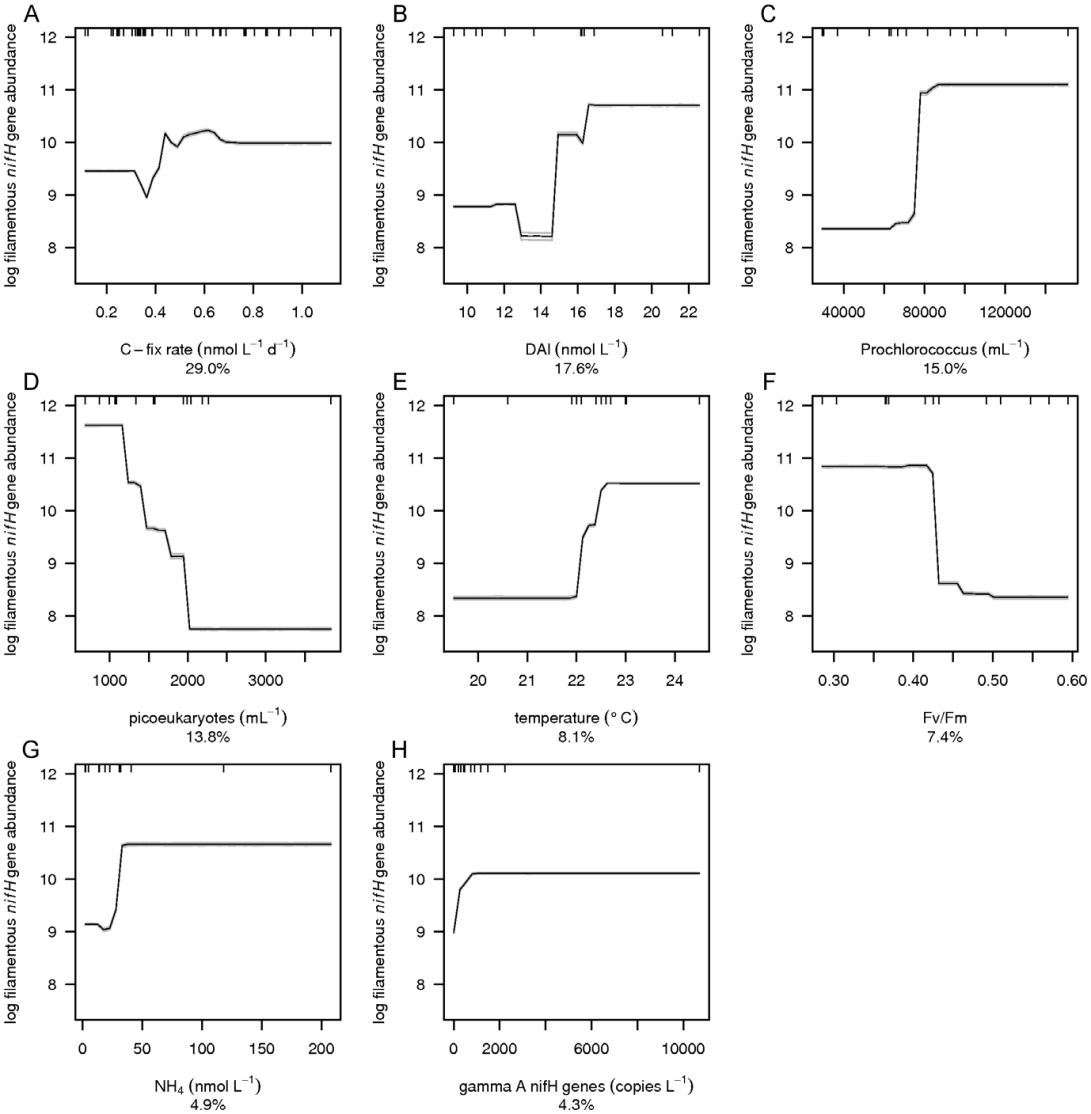
Mean partial dependence plots for the environmental variables describing the filamentous *nifH* gene abundance. Mean partial dependence plots with 95% confidence intervals (light grey, based on bootstrapping of 30 BRT runs and indicating robustness of model performance) for the 8 environmental variables which explain the variation in filamentous *nifH* gene abundance best. The 8 environmental variables are A) C-fix rate, B) DAl, C) *Prochloroccocus*, D) picoeukaryotes, E) F_v_/F_m_, F) NH_4_^+^, G) temperature and H) gamma A *nifH* genes. The y-axis is centered to have zero mean over the log distribution of the fitted filamentous *nifH* gene abundance. A common scale is used for the x-axis. Rug plots at inside top of plots show the distribution of sites across that variable, in deciles.

**Figure 8:**
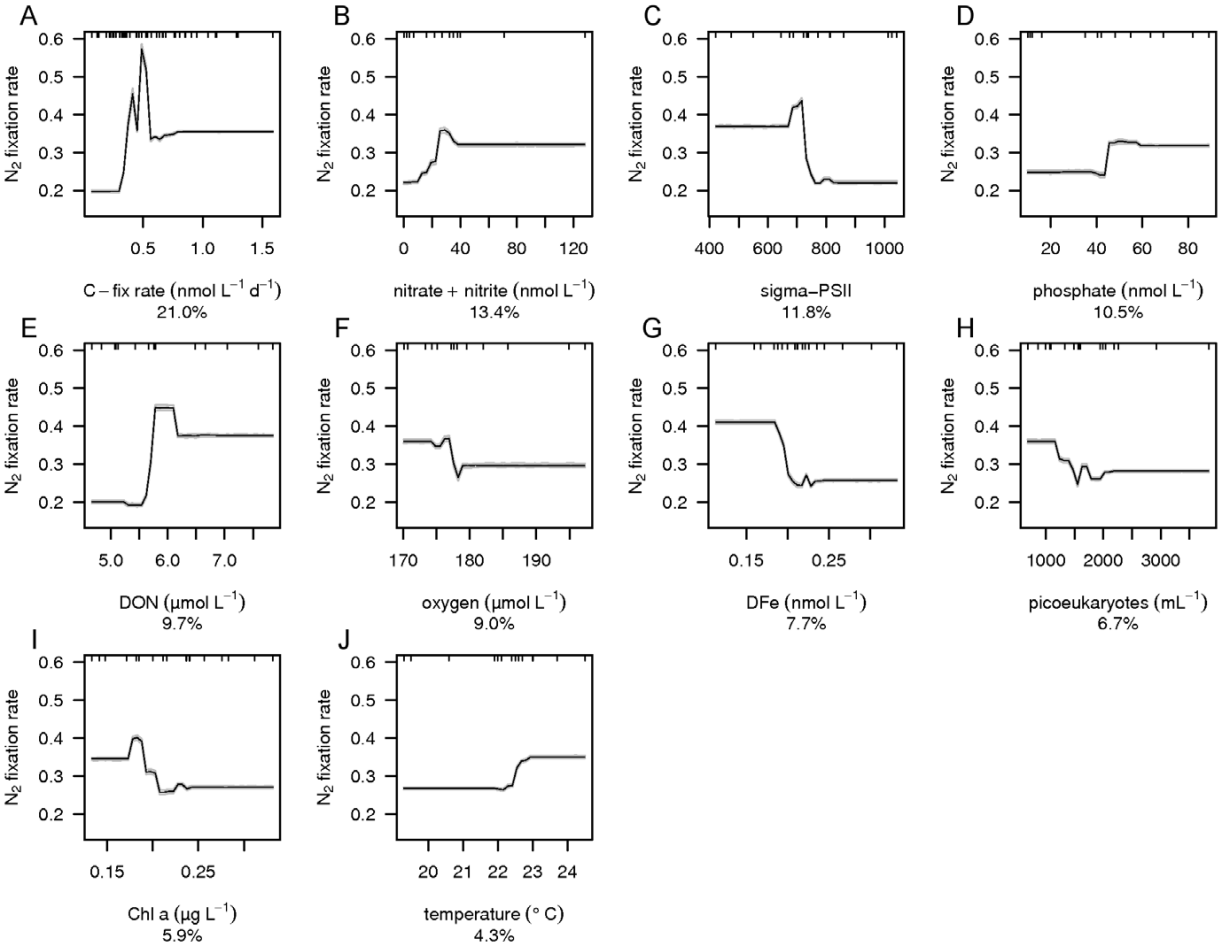
Mean partial dependence plots for the environmental variables describing the N_2_ fixation rates. Mean partial dependence plots with 95% confidence intervals (light grey, based on bootstrapping of 30 BRT runs and indicating robustness of model performance) for the 10 environmental variables which explain the variation in N_2_ fixation rates best. The environmental variables are A) C-fix rate, B) phosphate, C) nitrate + nitrite, D) sigma-PSII, E) DON, F) oxygen, G) DFe, H) picoeukaryotes, I) Chl *a*, and J) temperature. The y-axis is centered to have zero mean over the distribution of fitted N_2_ fixation rates. A common scale is used for the x-axis. Rug plots at inside top of plots show the distribution of sites across that variable, in deciles.

**Table 1:** Mean predictive performance of the BRT models and contributions of explanatory variables to the prediction of the N_2_ fixation rates, the total concentration of *nifH* genes, the concentration of *nifH* genes of filamentous cyanobacteria and Gamma A, a gamma-proteobacterium.

dependent vasriable	model characteristics		explanatory variables	Contribution%
total *nifH* genes	learning rate	0.05	Prochlorococcus	41.3±1.2
	tree complexity	3	Synechococcus	24.5±1.4
	trees fitted	2173±200	picoeukaryotes	19.8±1.2
	total dev.	5.334	DAl	9.4±1.1
	mean cv res. dev.	2.76±0.12	total Fe-binding ligands	3.1±0.7
	cv corr.	0.98±0.01	DFe	1.8±0.3
	D̂2	0.48		
Gamma A *nifH* genes	learning rate	0.01	Synechococcus	23.8±0.7
	tree complexity	3	Prochlorococcus	22.8±0.6
	trees fitted	3697±547	DON	17.3±0.8
	total dev.	8.221	picoeukaryotes	15.5±0.8
	mean cv res. dev.	3.59±0.08	Heterotr. bacteria	14.6±1.0
	cv corr.	0.94±0.01	Group A *nif* genes	6.2±0.8
	D̂2	0.56		
Filamentous *nifH* genes	learning rate	0.01	CO_2_ fixation rate	29.0±3.4
	tree complexity	3	DAl	17.6±2.3
	trees fitted	4185±354	Prochlorococcus	15.0±0.9
	total dev.	15.446	picoeukaryotes	13.8±1.1
	mean cv res. dev.	7.08±0.2	temp.	8.1±0.6
	cv corr.	0.53±0.01	F_v_/F_m_	7.4±0.6
	D̂2	0.54	NH_4_^+^	4.9±0.6
			Gamma A *nif* genes	4.3±0.6
N_2_ fixation rates	learning rate	0.05	CO_2_ fixation rate	21.0±2.4
	tree complexity	3	NO_3_^-^ + NO_2_^-^	13.4±1.5
	trees fitted	1820±632	PSII cross-section	11.8±1.0
	total dev.	0.081	phosphate	10.5±0.6
	mean cv res. dev.	0.053±0.003	DON	9.7±0.7
	cv corr.	0.47±0.02	oxygen	9.0±0.6
	D̂2	0.36	DFe	7.7±0.6
			picoeukaryotes	6.7±0.5
			Chl *a*	5.9±0.6
			temp.	4.3±0.6

The learning rate, tree complexity and number of trees fitted (trees fitted), as well as the total deviance for a saturated model (total dev.), are given for each model. Model performance measures, estimated using 30 model runs and 6-fold cross validation, included mean residual deviance and its standard error, the mean proportion of the total deviance explained (*D* ^2^) and the mean Pearson correlation (cv corr.) and its standard error.

The abundances of *Prochlorococcus* (Fig. 4m), *Synechococcus* (Fig. 4n) and picoeukaryotes (Fig. 4O) contributed strongly to the explanation of the distribution of the total *nifH* gene and gamma A *nifH* gene abundances (Fig. 5 and 6, Table 1). *Prochlorococcus* and picoeukaryotes were also important in explaining filamentous *nifH* gene abundance (Fig. 7). In all cases *nifH* genes were more abundant with higher abundance of *Prochlorococcus* and lower abundance of *Synechococcus* and picoeukaryotes. High *nifH* gene abundance coinciding with the non-N_2_ fixing *Prochlorococcus* abundance suggests co-occupation of the same environment. *Prochlorococcus* spp. have a very wide oceanic distribution and, like the majority of the oceanic N_2_-fixing organisms, thrive at enhanced temperatures with maximum abundances at 26–29°C [52]. *Prochlorococcus* as well as N_2_-fixing organisms are most abundant in, but not restricted to, oligotrophic waters while *Synechococcus* may have high abundances in nutrient-rich tropical environments [3], [52], [53].

The abundance of group A *nifH* genes (Fig. 4E) contributed 6% to the prediction of gamma A *nifH* gene abundance (Fig. 6), and gamma A *nifH* gene abundance contributed 4% to the prediction of filamentous *nifH* gene abundance (Fig. 7). As expected the positive relationship in both cases indicates that the different phyloptypes live side by side in the marine environment. However, *nifH* gene distributions of the group A, gamma A and filamentous diazotrophic phylotypes did not explain the N_2_ fixation rates (Fig. 8) indicating that the presence of *nifH* genes does not necessarily indicate an active metabolic pathway leading to N_2_ fixation. We further observed that heterotrophic prokaryote cell abundances (Fig. 4P) contributed 15% to the prediction of gamma A *nifH* gene abundance (Fig. 6).

Dissolved Al (Fig. 4L), a proxy for dust input, and consequently a proxy for the input of aeolian Fe and PO_4_^3-^ contributed 9% to the explanation of total *nifH* gene abundance and 18% to filamentous *nifH* gene abundance with higher *nifH* gene abundances at higher DAl concentrations (Fig. 5 and 7) [30], [54]. Our budget calculations (see above) suggest that DFe and PO_4_^3-^ did not limit primary production or N_2_ fixation. However, as DAl represents the cumulative effects of dust inputs rather than signifying individual short-term dust events, the general distribution of N_2_ fixing organisms may be determined by general patterns of dust inputs as also shown by Langlois et al. (2008). Contributions of DFe and total Fe-binding ligand concentrations (Fig. 4U), important to keep aeolian Fe in solution [30], were relatively small in explaining total *nifH* gene abundance and did not explain gamma A and filamentous *nifH* gene abundance. The lower DFe concentrations found at higher total *nifH* gene abundance may be the result of biological uptake. Photosynthesis as well as N_2_ fixation requires Fe, with N_2_ fixation requiring about 5–10 times more Fe than NO_3_^-^ utilization [55]. BRT showed that both higher N_2_ fixation rates and higher filamentous *nifH* gene abundance coincided with a higher CO_2_ fixation rate altogether resulting in relative high demands for Fe with consequently lower DFe concentrations at higher total *nifH* gene abundances (Fig. 7 and 8).

The CO_2_ fixation rate was the main contributor in explaining filamentous *nifH* gene abundance (29%) as well as N_2_ fixation rates (21%) (Fig. 7 and 8). Considering that the N_2_ fixation rate contributed up to 5% of the N required for new production, the high contribution of the CO_2_ fixation rates in predicting the distribution of filamentous *nifH* gene abundance and N_2_ fixation rates may be a consequence of fixed N release into the marine environment. Alternatively, environmental circumstances that promote high CO_2_ fixation rates also promote the presence of filamentous diazotrophs resulting in higher N_2_ fixation rates in our incubations. Also temperature contributed to the filamentous *nifH* gene abundance as well as N_2_ fixation rates, with higher abundance and rates in waters with temperatures above 22–23°C (∼8 and 4% contribution respectively). The similarity in the model response as shown by the partial dependence plots of CO_2_ fixation rate and temperature for the filamentous *nifH* gene abundance and N_2_ fixation rates suggests that the filamentous phylotypes may have been responsible for the main part of the N_2_ fixation rates. The low contribution of temperature to explaining N_2_ fixation is perhaps surprising as it has been shown that N_2_ fixation by *Trichodesmium* occurs at temperatures between 20°C and 34°C, with an optimum of 27°C [13], [14]. The surface water temperatures encountered during the cruise varied between 18 and 25°C, hence below the reported optimum values. The BRT model results suggest a step-up in N_2_ fixation between 22 and 23°C coinciding with the optimum temperature for the presence of group A and filamentous cyanobacteria (Langlois et al. 2008). Lower oxygen solubility in combination with faster metabolic processes such as respiration, facilitating oxygen scavenging, may underlie this high temperature preference [3], [14], [56], [57]. This idea concurs with our finding that N_2_ fixation rates varied negatively with oxygen concentrations.

N_2_ fixation was higher at lower dissolved oxygen concentrations (9% contribution) (Fig. 4V and 8). This is likely due to the oxygen sensitive nature of the nitrogenase enzyme, resulting in a decrease in nitrogenase activity with increasing oxygen concentrations [14], [58], [59]. While *Trichodesmium* and most unicellular microorganisms grow and fix N_2_ under fully aerobic conditions, the BRT analysis predicts that lower environmental concentrations of oxygen may be advantageous for N_2_ fixation.

The photophysiological variables as F_v_/F_m_ (7%, Fig. 4S) and σ_PSII_ (12%, Fig. 4R) both contributed to the prediction of filamentous *nifH* gene abundance and N_2_ fixation, respectively. F_v_/F_m_, the photochemical efficiency, and σ_PSII_, the photosystem II effective absorption cross section, both give information about effects of physiological stress on the photosystem and changes in community structure [60]. The magnitude in variability of F_v_/F_m_ and σ_PSII_ due to changes in phytoplankton community structure often exceeds that induced by nutrient limitation [60]. As discussed above, the most likely limiting nutrient in the vicinity of the Cape Verdes was nitrogen. Nitrogen limitation may cause physiological stress on the photosystem in the overall community and strengthen the competitiveness of N_2_-fixing organisms resulting in the observed negative relationships between the photophysiological variables and filamentous *nifH* gene abundance and N_2_ fixation rates (Fig. 7 and 8). Alternatively, the change of filamentous *nifH* gene abundance and N_2_ fixation with these photophysiological variables may represent a change in community structure. Chl *a* concentrations, contributing 6% to predicting N_2_ fixation rates (Fig. 4Q), were low at high N_2_ fixation rates and may also have been the result of changes in community structure [61] (Fig. 8).

The distribution of diazotrophs as well as the N_2_ fixation pathway is regulated by the presence of nitrogen containing compounds [3], [43]. DON (Fig. 4I) contributed 17% to the prediction of gamma A *nifH* gene abundance, NH_4_^+^ (Fig. 4H) contributed 5% to the prediction of filamentous *nifH* gene abundance, while NO_3_^-^ (Fig. 4G), and DON contributed 13% and 10%, respectively, to the prediction of N_2_ fixation rates (Fig. 6–8).

In the presence of micro-molar concentrations of NO_3_^-^, a biochemical down regulation of the N_2_ fixation pathway occurs [19], [20], [43]. However, at relatively low concentrations (300–400 nmol L^−1^ NO_3_^-^), N_2_ fixation in *Trichodesmium* spp. has been reported to recover [20]. Overall, the NO_3_^-^ pool in our experiments was <130 nmol L^−1^ (and total DIN <350 nmol L^−1^), below NO_3_^-^ concentrations at which N_2_ fixation rates would be inhibited. Although an increase in N_2_ fixation rates with increasing NO_3_^-^ up to 30 nmol L^−1^, as suggested by the BRT analysis (Fig. 8), may appear surprising, we know that NO_3_^-^ concentrations were low in our experiments and may have been quickly exhausted. It is known that *Trichodesmium* prefers to utilize NH_4_^+^ and NO_3_^-^ over N_2_ fixation [20], [21]. As supported by the BRT analysis that predicts higher filamentous *nifH* gene abundance at higher NH_4_^+^ concentrations, more diazotrophs such as *Trichodesmium* may have been present in the seawater from the incubations with slightly higher NH_4_^+^ and NO_3_^-^ concentrations. As a consequence, with diminishing concentrations of NH_4_^+^ and NO_3_^-^ the higher abundance of diazotrophs may have resulted in the fixation of more N_2_. The positive relationship between filamentous *nifH* gene abundance and NH_4_^+^ may also be explained by the release, and subsequent regeneration, of amino acids and DON by *Trichodesmium* during N_2_ fixation [62], [63].

DON contributed 17% to the prediction of gamma A *nifH* gene abundance and 10% to the prediction of N_2_ fixation rates. DON can be produced as well as utilized by diazotrophs [43], [62]. Gamma A *nifH* gene abundance was higher at lower DON concentrations consistent with the hypothesis that N depleted environments promote the growth of N_2_-fixing organisms. The positive relationship between N_2_ fixation rate and DON could be explained by the exudation of fixed N in the form of, for example, amino acids [62]. Both explanations are plausible considering that enhanced gamma A *nifH* gene abundance does not mean that N_2_ fixation is active.

PO_4_^3-^ (Fig 4J) contributed 11% to explaining N_2_ fixation rates. PO_4_^3-^ forms an important nutrient that potentially limits N_2_ fixation [18]. Fitted values predicted that N_2_ fixation increased at ca. 40 nmol L^−1^ PO_4_^3-^ (Fig. 8). This suggests that N_2_ fixation may be released from PO_4_^3-^ limitation above a PO_4_^3-^ concentration of ca. 40 nmol L^−1^. Concentrations this low are common in the central and Western North Atlantic [16], [46], [64]. With the exception of the most northern sites our study area generally had PO_4_^3-^ concentrations exceeding this limit (Fig. 4J).

It is clear that no single factor controls the distribution of diazotrophs and N_2_ fixation but that these are determined by a combination of variables. This is especially true for areas and seasons where no nutrients, trace metals or other environmental parameters are directly limiting or inhibiting diazotrophy. Not all environmental variables considered in this study may have been causal for N_2_ fixation and the distribution of diazotrophs. However, from experimental and field studies it is known that a-biotic environmental variables such as NO_3_^-^ + NO_2_^-^, DON, Fe, PO_4_^3-^, temperature and oxygen directly affect N_2_ fixation in the North Atlantic Ocean [16], [17], [18], [21], [56], [57]. In this study, BRT analysis showed that these a-biotic environmental variables all contributed to determining N_2_ fixation rates and that some of them also affected the distribution of gamma A, filamentous and total *nifH* gene abundances in the eastern tropical and sub-tropical North Atlantic Ocean.

### Potential consequences of climate warming on N_2_ fixation

Identification of the a-biotic environmental variables that have the potential to limit or inhibit N_2_ fixation allows us to assess how a change in these environmental variables due to climate warming may affect future N_2_ fixation rates. The warming of the earth system will result in enhanced sea surface temperature (SST), with predicted enhanced deposition of Saharan dust to the surface oceans [65]. N_2_ fixation rates in the eastern tropical and sub-tropical North Atlantic Ocean are likely to be affected by enhanced SST, reduced oxygen concentrations, reduced nutrient supply and an increase in light availability due to enhanced water column stratification. According to our results N_2_ fixation rates will increase at lower oxygen concentrations and higher temperatures and are thus consistent with previous work. In addition, N_2_ fixation may be promoted by an increase in light availability, an enhanced CO_2_ concentration and changes in N/P stoichiometry induced by shifts in the phytoplankton community [66], [67], [68].

However, in the eastern tropical and subtropical North Atlantic an increase in N_2_ fixation may be hampered by PO_4_^3-^ limitation and, although not shown in this study, potentially by Fe limitation, should the availability of either element decrease. Due to future enhanced stratification, we may experience a decrease in upward mixing into the surface waters of deeper waters with excess phosphate (excPO_4_^3-^ = PO_4_^3-^ - (NO_3_^-^ + NO_2_^-^)/16; excPO_4_^3-^ = 14 e ^0.02 x Apparent Oxygen Utilization^, R^2^ = 0.71, p<0.001, n = 25) and high concentrations of Fe, derived from the oxygen minimum zone situated south and west of the Cape Verde. Increased atmospheric Fe and PO_4_^3-^ inputs from the Sahara, may potentially off set the reduced deep water supply; the atmospheric inputs are however more sporadic. As a consequence, the balance between a reduced supply of nutrients due to stratification and an enhanced nutrient input with increasing deposition of Saharan dust may ultimately determine the consequences of climate change for N_2_ fixation in the eastern tropical and sub-tropical North Atlantic.

## References

[1] Capone DG (2001). Marine nitrogen fixation: what's the fuss?. Curr Opin Microbiol.

[2] Karl DM, Letelier RM (2008). Nitrogen fixation-enhanced carbon sequestration in low nitrate, low chlorophyll seascapes.. Mar Ecol Progr Ser.

[3] Langlois RJ, Hummer D, LaRoche J (2008). Abundances and distributions of the dominant nifH phylotypes in the Northern Atlantic Ocean.. Appl Environ Microb.

[4] Capone DG, Burns JA, Montoya JP, Subramaniam A, Mahaffey C (2005). Nitrogen fixation by Trichodesmium spp.: An important source of new nitrogen to the tropical and subtropical North Atlantic Ocean.. Global Biogeochem Cycles.

[5] Langlois RJ, LaRoche J, Raab PA (2005). Diazotrophic diversity and distribution in the tropical and subtropical Atlantic ocean.. Appl Environ Microb.

[6] Zehr JP, Waterbury JB, Turner PJ, Montoya JP, Omoregie E (2001). Unicellular cyanobacteria fix N-2 in the subtropical North Pacific Ocean.. Nature.

[7] Zehr JP, Mellon MT, Zani S (1998). New nitrogen-fixing microorganisms detected in oligotrophic oceans by amplification of nitrogenase (nifH) genes.. Appl Environ Microb.

[8] Falcon LI, Cipriano F, Chistoserdov AY, Carpenter EJ (2002). Diversity of diazotrophic unicellular cyanobacteria in the tropical North Atlantic Ocean.. Appl Environ Microb.

[9] Montoya JP, Holl CM, Zehr JP, Hansen A, Villareal TA (2004). High rates of N-2 fixation by unicellular diazotrophs in the oligotrophic Pacific Ocean.. Nature.

[10] Montoya JP, Voss M, Capone DG (2007). Spatial variation in N-2-fixation rate and diazotroph activity in the Tropical Atlantic.. Biogeosciences.

[11] Voss M, Croot P, Lochte K, Mills M, Peeken I (2004). Patterns of nitrogen fixation along 10N in the tropical Atlantic.. Geophys Res Lett.

[12] Staal M, Hekkert ST, Brummer GJ, Veldhuis M, Sikkens C (2007). Nitrogen fixation along a north-south transect in the eastern Atlantic Ocean.. Limnol Oceanogr.

[13] Breitbarth E, Oschlies A, LaRoche J (2007). Physiological constraints on the global distribution of Trichodesmium - effect of temperature on diazotrophy.. Biogeosciences.

[14] LaRoche J, Breitbarth E (2005). Importance of the diazotrophs as a source of new nitrogen in the ocean.. J Sea Res.

[15] Berman-Frank I, Cullen JT, Shaked Y, Sherrell RM, Falkowski PG (2001). Iron availability, cellular iron quotas, and nitrogen fixation in Trichodesmium.. Limnol Oceanogr.

[16] Moore CM, Mills MM, Achterberg EP, Geider RJ, LaRoche J (2009). Large-scale distribution of Atlantic nitrogen fixation controlled by iron availability.. Nature Geosci.

[17] Sanudo-Wilhelmy SA, Kustka AB, Gobler CJ, Hutchins DA, Yang M (2001). Phosphorus limitation of nitrogen fixation by Trichodesmium in the central Atlantic Ocean.. Nature.

[18] Mills MM, Ridame C, Davey M, La Roche J, Geider RJ (2004). Iron and phosphorus co-limit nitrogen fixation in the eastern tropical North Atlantic.. Nature.

[19] Fu F-X, Bell PRF (2003). Factors affecting N2 fixation by the cyanobacterium Trichodesmium sp. GBRTRLI101.. Fems Microbiol Ecol.

[20] Holl CM, Montoya JP (2005). Interactions between nitrate uptake and nitrogen fixation in continuous cultures of the marine diazotroph Trichodesmium (Cyanobacteria).. J Phycol.

[21] Mulholland MR, Capone DG (1999). Nitrogen fixation, uptake and metabolism in natural and cultured populations of Trichodesmium spp.. Mar Ecol Progr Ser.

[22] Holl CM, Waite AM, Pesant S, Thompson PA, Montoya JP (2007). Unicellular diazotrophy as a source of nitrogen to Leeuwin Current coastal eddies.. Deep Sea Res II.

[23] Needoba JA, Foster RA, Sakamoto C, Zehr JP, Johnson KS (2007). Nitrogen fixation by unicellular diazotrophic cyanobacteria in the temperate oligotrophic North Pacific Ocean.. Limnol Oceanogr.

[24] Chiapello I, Bergametti G, Gomes L, Chatenet B, Dulac F (1995). An additional low layer transport of Sahelian and Saharan dust over the North-Eastern Tropical Atlantic.. Geophys Res Lett.

[25] Mahaffey C, Michaels AF, Capone DG (2005). The conundrum of marine N-2 fixation.. Am J Sci.

[26] Turk KA, Rees AP, Zehr JP, Pereira N, Swift P (2011). Nitrogen fixation and nitrogenase (nifH) expression in tropical waters of the eastern North Atlantic.. ISME J.

[27] Montoya JP, Voss M, Kahler P, Capone DG (1996). A simple, high-precision, high-sensitivity tracer assay for N-2 fixation.. Appl Environ Microb.

[28] Avak H, Fry B (1999). EA-IRMS: Precise and Accurate Measurement of δ^15^N on <10 ug N.. Finnigan MAT Application Flash Report.

[29] Hamme RC, Emerson SR (2004). The solubility of neon, nitrogen and argon in distilled water and seawater.. Deep Sea Res I.

[30] Rijkenberg MJA, Powell CF, Dall'Osto M, Nielsdottir MC, Patey MD (2008). Changes in iron speciation following a Saharan dust event in the tropical North Atlantic Ocean.. Mar Chem.

[31] Hydes DJ, Liss PS (1976). Fluorimetric method for determination of low concentrations of dissolved aluminum in natural waters.. Analyst.

[32] Patey MD, Rijkenberg MJA, Statham PJ, Stinchcombe MC, Achterberg EP (2008). Determination of nitrate and phosphate in seawater at nanomolar concentrations.. Trac-Trend Anal Chem.

[33] Kirkwood D (1996). Nutrients: Practical notes on their determination in sea water, *ICES Techniques in Marine Environmental Sciences*.. International Council for the Exploration of the Sea.

[34] Kerouel R, Aminot A (1997). Fluorometric determination of ammonia in sea and estuarine waters by direct segmented flow analysis.. Mar Chem.

[35] Badr ESA, Achterberg EP, Tappin AD, Hill SJ, Braungardt CB (2003). Determination of dissolved organic nitrogen in natural waters using high-temperature catalytic oxidation.. Trac-Trend Anal Chem.

[36] Zubkov MV, Sleigh MA, Burkill PH, Leakey RJG (2000). Picoplankton community structure on the Atlantic Meridional Transect: a comparison between seasons.. Prog Oceanogr.

[37] Atallah ZK, Bae J, Jansky SH, Rouse DI, Stevenson WR (2007). Multiplex real-time quantitative PCR to detect and quantify Verticillium dahliae colonization in potato lines that differ in response to Verticillium wilt.. Phytopathology.

[38] Elith J, Leathwick JR, Hastie T (2008). A working guide to boosted regression trees.. J Anim Ecol.

[39] Friedman J, Hastie T, Tibshirani R (2000). Additive logistic regression: A statistical view of boosting.. Ann Stat.

[40] Ridgeway G (2004). gbm: generalized boosted regression models.. R package, version.

[41] Stramma L, Huttl S, Schafstall J (2005). Water masses and currents in the upper tropical northeast Atlantic off northwest Africa.. J Geophys Res-Oceans.

[42] Falcon LI, Carpenter EJ, Cipriano F, Bergman B, Capone DG (2004). N-2 fixation by unicellular bacterioplankton from the Atlantic and Pacific oceans: Phylogeny and in situ rates.. Appl Environ Microb.

[43] Mulholland MR, Ohki K, Capone DG (2001). Nutrient controls on nitrogen uptake and metabolism by natural populations and cultures of Trichodesmium (Cyanobacteria).. J Phycol.

[44] Mulholland MR, Capone DG (2000). The nitrogen physiology of the marine N2-fixing cyanobacteria Trichodesmium spp.. Trends Plant Sci.

[45] Mulholland MR, Ohki K, Capone DG (1999). Nitrogen utilization and metabolism relative to patterns of N-2 fixation in cultures of Trichodesmium NIBB1067.. J Phycol.

[46] Wu JF, Sunda W, Boyle EA, Karl DM (2000). Phosphate depletion in the western North Atlantic Ocean.. Science.

[47] Ho TY, Quigg A, Finkel ZV, Milligan AJ, Wyman K (2003). The elemental composition of some marine phytoplankton.. J Phycol.

[48] Kustka A, Carpenter EJ, Sanudo-Wilhelmy SA (2002). Iron and marine nitrogen fixation: progress and future directions.. Res Microbiol.

[49] de Baar HJW, Gerringa LJA, Laan P, Timmermans KR (2008). Efficiency of carbon removal per added iron in ocean iron fertilization.. Mar Ecol Prog Ser.

[50] Rijkenberg MJA, Gerringa LJA, Timmermans KR, Fischer AC, Kroon KJ (2008). Enhancement of the reactive iron pool by marine diatoms.. Mar Chem.

[51] Maldonado MT, Strzepek RF, Sander S, Boyd PW (2005). Acquisition of iron bound to strong organic complexes, with different Fe binding groups and photochemical reactivities, by plankton communities in Fe-limited subantarctic waters.. Global Biogeochem Cycles.

[52] Partensky F, Hess WR, Vaulot D (1999). Prochlorococcus, a marine photosynthetic prokaryote of global significance.. Microbiol Mol Biol Rev.

[53] Zubkov MV, Fuchs BM, Tarran GA, Burkill PH, Amann R (2003). High rate of uptake of organic nitrogen compounds by Prochlorococcus cyanobacteria as a key to their dominance in oligotrophic oceanic waters.. Applied And Environmental Microbiology.

[54] Bartoli G, Migon C, Losno R (2005). Atmospheric input of dissolved inorganic phosphorus and silicon to the coastal northwestern Mediterranean Sea: Fluxes, variability and possible impact on phytoplankton dynamics.. Deep Sea Res I.

[55] Kustka AB, Sanudo-Wilhelmy SA, Carpenter EJ, Capone D, Burns J (2003). Iron requirements for dinitrogen- and ammonium-supported growth in cultures of Trichodesmium (IMS 101): Comparison with nitrogen fixation rates and iron: carbon ratios of field populations.. Limnol Oceanogr.

[56] Staal M, Meysman FJR, Stal LJ (2003). Temperature excludes N-2-fixing heterocystous cyanobacteria in the tropical oceans.. Nature.

[57] Stal LJ (2009). Is the distribution of nitrogen-fixing cyanobacteria in the oceans related to temperature?. Environ Microbiol.

[58] Berman-Frank I, Lundgren P, Chen YB, Kupper H, Kolber Z (2001). Segregation of nitrogen fixation and oxygenic photosynthesis in the marine cyanobacterium Trichodesmium.. Science.

[59] Staal M, Rabouille S, Stal LJ (2007). On the role of oxygen for nitrogen fixation in the marine cyanobacterium Trichodesmium sp.. Environ Microbiol.

[60] Suggett DJ, Moore CM, Hickman AE, Geider RJ (2009). Interpretation of fast repetition rate (FRR) fluorescence: signatures of phytoplankton community structure versus physiological state.. Mar Ecol Progr Ser.

[61] Hirata T, Hardman-Mountford NJ, Brewin RJW, Aiken J, Barlow R (2011). Synoptic relationships between surface Chlorophyll-a and diagnostic pigments specific to phytoplankton functional types.. Biogeosciences.

[62] Capone DG, Ferrier MD, Carpenter EJ (1994). Amino acid cycling in colonies of the planktonic marine cyanobacterium *Trichodesmium thiebautii*.. Appl Environ Microb.

[63] Mulholland MR, Bronk DA, Capone DG (2004). Dinitrogen fixation and release of ammonium and dissolved organic nitrogen by Trichodesmium IMS101.. Aquat Microb Ecol.

[64] Mather RL, Reynolds SE, Wolff GA, Williams RG, Torres-Valdes S (2008). Phosphorus cycling in the North and South Atlantic Ocean subtropical gyres.. Nature Geosci.

[65] Mahowald NM, Engelstaedter S, Luo C, Sealy A, Artaxo P (2009). Atmospheric iron deposition: global distribution, variability, and human perturbations.. Annu Rev Mar Sci.

[66] Hutchins DA, Fu F-X, Zhang Y, Warner ME, Feng Y (2007). CO2 control of Trichodesmium N2 fixation, photosynthesis, growth rates, and elemental ratios: Implications for past, present, and future ocean biogeochemistry.. Limnol Oceanogr.

[67] Mills MM, Arrigo KR (2010). Magnitude of oceanic nitrogen fixation influenced by the nutrient uptake ratio of phytoplankton.. Nature Geosci.

[68] Tagliabue A, Bopp L, Gehlen M (2011). The response of marine carbon and nutrient cycles to ocean acidification: Large uncertainties related to phytoplankton physiological assumptions.. Global Biogeochem Cycles.

